# Bibliometric analysis and visualization of top papers in dentistry from 2012 to 2022 based on essential science indicators

**DOI:** 10.1002/cre2.832

**Published:** 2024-02-03

**Authors:** Amene Taghdisi kashani, Zahra Batooli, Mostafa Mozafari

**Affiliations:** ^1^ Department of Orthodontics, Dentistry Faculty Kashan University of Medical Sciences Kashan Iran; ^2^ Social Determinants of Health (SDH) Research Center Kashan University of Medical Sciences Kashan Iran

**Keywords:** bibliometric analysis, dentistry, essential science indicators, top papers

## Abstract

**Objectives:**

This study aims to analyze and visualize the top dental papers from 2012 to 2022 using data from essential science indicators (ESIs).

**Materials and Methods:**

The present study was conducted using library and retrospective bibliometric analysis methods. Additionally, a science map has been created. Web of Science was searched on January 18, 2023. The results were limited to the top papers in ESIs. The bibliometric information of the top papers was evaluated. Next, the VOSviewer was used to perform a co‐occurrence analysis and visualize data.

**Results:**

The findings showed that *Mariano Belén Sanz* and *Maurizio S. Tonetti* were the top two authors. The University of London and the University of Bern had the highest number of articles. These articles were published in 55 journals. According to the analysis of keyword co‐occurrence, the most frequently used keywords in the field of dentistry include “periodontitis,” “dental implants,” “periodontal disease,” “mechanical properties,” “peri‐implantitis,” “oral health,” “dental caries,” “dental materials,” “3D printing.”

**Conclusion:**

The findings of this study enable readers to pinpoint the authors, organizations, countries, and journals that have made the biggest contributions to the list of the most often cited dentistry papers. In medicine, bibliometric citation analysis is frequently used to assist researchers in learning the fundamentals of a subject and pinpoint subtopics of broad interest for additional study.

## 1 INTRODUCTION

The field of dentistry and related sciences are directly connected to the lives and health of humans. Given the high cost of education and research in this field, proper planning is necessary (Daryakenari & Batooli, 2022). Dentistry encompasses ten branches, which include oral and dental disease diagnosis, oral pathology, oral and facial imaging, restorative dentistry, root canal treatment, oral and facial prosthetics, pediatric dentistry, orthodontics, gum surgery, and oral and facial surgery. Each subtopic of dentistry can play a significant role in the scientific advancement of a country's dental field (Sheikhshoaei et al., 2021). Measuring various aspects of research in these subject areas can be effective in planning future studies and facilitating extensive development in various fields of dental sciences. It can also aid in optimizing the allocation of budget and resources, ultimately improving the quality and quantity of the products in this field (Daryakenari & Batooli, 2022).

The evaluation of scientific production provides an effective tool for policy‐making and planning, as well as helping to identify weaknesses and deficiencies in scientific output (Bazrafshan & Mostafavi, 2011). On the other hand, evaluating highly cited articles from different fields can help professionals gain a better understanding of the nature of those fields. While it is important to note that citations alone do not serve as a definitive measure of quality, they do indicate the level of response generated by a scientist's work within the scientific community. Typically, high‐quality work tends to elicit a greater number of citations from fellow scientists compared to lower‐quality work (Bornmann & Daniel, 2008).

Due to the importance of this topic, several research studies have been conducted in recent years to review highly cited articles in the field of dentistry. Some studies have focused on the bibliometric evaluation of articles in the dental field, including those related to COVID‐19 from a dental research perspective (Jacimovic et al., 2021), and dental Anxiety (Yeung, 2023). Some studies have also evaluated highly cited articles on various dental topics, including prosthetics (Praveen et al., 2020), orthodontics (Hui et al., 2013), root canals (Fardi et al., 2011), periodontology (Ahmad, Asif, et al., 2020), and oral leukoplakia (Liu et al., 2019). The evaluation of 33 articles, in each of which 100 highly cited articles from different fields of dentistry have been evaluated, is another study in this connection (Daryakenari & Batooli, 2022).

The essential science indicators (ESI) is a subset of the Science Citation Index that compiles articles that have been among the top 1% of highly cited papers over the past decade (Batooli, 2017). Meanwhile, the highly cited articles (top 1%) in the field of dentistry and related sciences have not been evaluated in the science citation database in the last 10 years. Therefore, the purpose of this study is to evaluate and visualize the highly cited dental papers from 2012 to 2022 using ESIs. The results of this study provide dental specialists with the opportunity to familiarize themselves with authors, countries, institutions, and core journals, as well as influential and interesting topics in the field of dentistry.

## 2 MATERIALS AND METHOD

This research analyzes the bibliometric aspects of highly cited dental papers from 2012 to 2022 based on the Web of Science (WoS). The present study is applied in terms of purpose and was conducted using library and retrospective bibliometric analysis methods. Additionally, a science map has been created. One of the techniques used in drawing science maps is the visualization of similarities (VOS). VOSviewer is a software tool used for bibliometric analysis (Van Eck & Waltman, 2010). A large number of studies have used this software (Mattos et al., 2021; Mayta‐Tovalino, 2022; Yu & Chang, 2022). This software is a tool for constructing and visualizing bibliometric networks, such as scientific publications, researchers, or articles. Each network can be created based on citation, bibliographic pairs, co‐citation, or coauthorship relations (Vosviewer, 2023).

To conduct bibliometric analysis, the bibliometrix R‐package was utilized. Bibliometrix is a unique open‐source tool for conducting comprehensive science mapping analysis. It supports a recommended workflow for conducting bibliometric analyses. As it is programmed in R, the proposed tool is flexible and can be easily upgraded and integrated with other statistical R‐packages. It is therefore useful in a constantly evolving field like bibliometrics (Aria & Cuccurullo, 2017). In this study, the software was utilized to create a map illustrating scientific cooperation among countries, as well as a word cloud map.

Research areas constitute a subject categorization scheme that is shared by all WoS product databases. Journals and books included in the WoS Core Collection are categorized under at least one WoS category. Each WoS category is mapped to a specific research area (WoS, 2020). In this research, articles in the field of dentistry were retrieved by searching the “research areas” field using the following search query: “SU = (DENTISTRY, ORAL SURGERY, AND MEDICINE).”

The search results showed that as of January 18, 2023, 492,754 articles in the field of dentistry have been indexed in this database. Then the results were limited to the top papers in ESIs). Top papers are a small group of articles that have received a significant number of citations. Next, the bibliometric information of the articles, such as publication year, author, country, journal, article type, publisher, citation count, citation density (which measures the relative impact of the article regardless of its publication year), and subject area, were evaluated.

To disambiguate certain terms and correct any transcription or indexing errors, the names of authors were manually refined and normalized. To address the issue of authors' names being written in different forms, a thesaurus was created to standardize all names. For example, chapple, i. l. c., chapple, iain, and chapple, iain l. c. All of which were identified as chapple, iain l. c. in the thesaurus. To conduct a co‐occurrence analysis, the keywords extracted from the reviewed articles were carefully examined and standardized to ensure consistency. This involved selecting one keyword from a list of synonymous options and replacing certain words to achieve consistency. Additionally, the singular and plural forms of words were standardized to a single form. When keywords appeared in both full and abbreviated forms, the full phrase was chosen as the selected keyword. Once the keywords were standardized, a co‐occurrence map was generated using VOSviewer.

## 3 RESULTS

### 3.1 All articles in the field of dentistry

**Figure 1:**
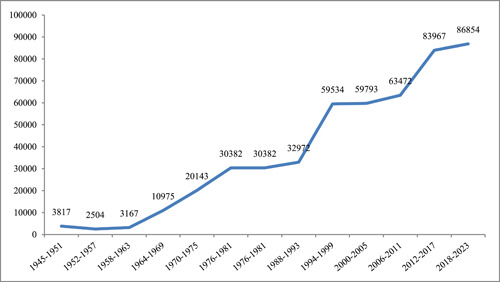
Dentistry articles by year.

The findings showed that 495,648 articles in the field of dentistry have been indexed in WoS (Figure 1).

Based on Figure 1, it can be observed that a significant proportion of articles, approximately 20%, have been published since 2017. The number of articles in the field of dentistry has been steadily increasing in recent years. Nine authors have been identified as the most prolific contributors in this field, each having authored over 600 articles.

*David Henry Pashley* from *Augusta University* and *Crispian Scully* from *University College London* top the list with an impressive 797 articles each. The *University of London* had the highest number of articles, with 12,587 (2.5%). The second and third positions were held by the *Universidade de São Paulo* and the *University of California System*, respectively.

Authors from 210 different countries contributed to the articles, with the *United States* being responsible for 26.2% of them. Following the *United States*, *England* contributed 7.5% of the articles, while *Brazil* contributed 6.9%. The reviewed articles spanned across 71 subject categories in the WoS database. “Dentistry, Oral Surgery, and Medicine” was the most prevalent subject category, with all articles falling under this category. “Surgery” (4.9%), “Oncology” (1.78%), and “Materials Science Biomaterials” (1.72%) were the next most common subject categories.

The analysis revealed that a total of 495,648 articles have been published in 692 journals. The *Journal of Dental Research* has the highest number of articles, with 102,238 articles (20.627%). The *British Dental Journal* and the *Journal of the American Dental Association* closely follow behind. *Elsevier* is the leading publisher with the highest number of articles, totaling 104,375 (21.05%). *Wiley* Publications and the *American Association for Dental Research* are ranked second and third, respectively.

### 3.2 Highly cited articles in the field of dentistry

After analyzing 495,648 articles in the field of dentistry, we found that only 292 articles were recognized as top papers. Nearly half of these articles were published in 2018 or later.

#### 3.2.1 Author

In terms of authorship, a total of 1341 authors contributed to these top papers, with only 21 authors responsible for producing 50% of them. Table 1 displays the authors who have written more than eight articles.

**Table 1:** Authors with more than eight top articles in the field of dentistry.

Author	No of articles	H‐index
Sanz, Mariano Belén	13	74
Tonetti, Maurizio S.	12	87
Chapple, Ian L.C.	11	61
Genco, Robert J.	11	104
Jepsen, Sören	8	60
Derks, Jan	8	23
Marcenes, Wagner Segura	8	66

**Figure 2:**
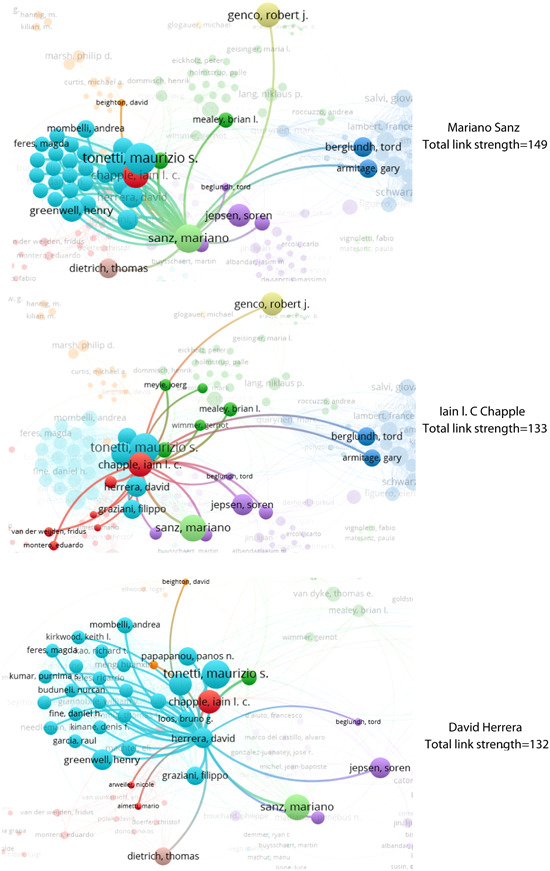
Top three authors by total link strength.

Table 1 shows that *Mariano Belén Sanz* from Spain, with 13 articles, and *Maurizio S. Tonetti* from China, with 12 articles, are the top two authors. Out of 1341 authors, only 324 had scientific collaborations with one another. Figure 2 displays the three authors with the highest total link strength.

**Figure 3:**
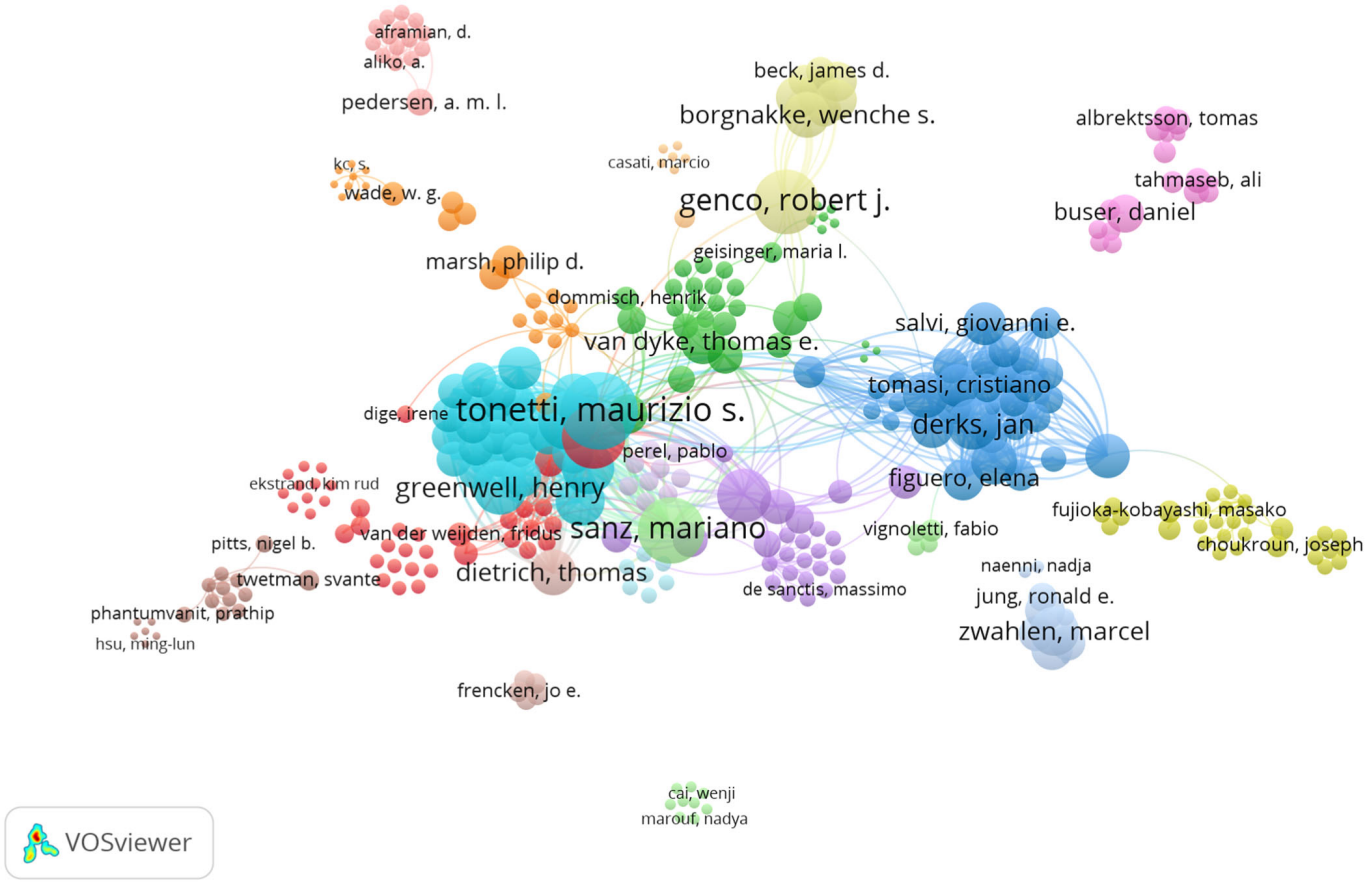
Authors in terms of the number of citations.

The results indicate that *Maurizio S. Tonetti*, *Filippo Graziani*, and *Soren Jepsen* are among the top authors, with total link strengths of 128, 106, and 101, respectively. Figure 3 illustrates the authors' status based on the number of citations they have received.

Figure 3 shows that *Maurizio S. Tonetti* is ranked first with 12 articles and 5684 citations, followed by *Mariano Sanz* with 13 articles and 4298 citations, and *Robert J. Genco* with 11 articles and 4298 citations.

#### 3.2.2 Country

**Figure 4:**
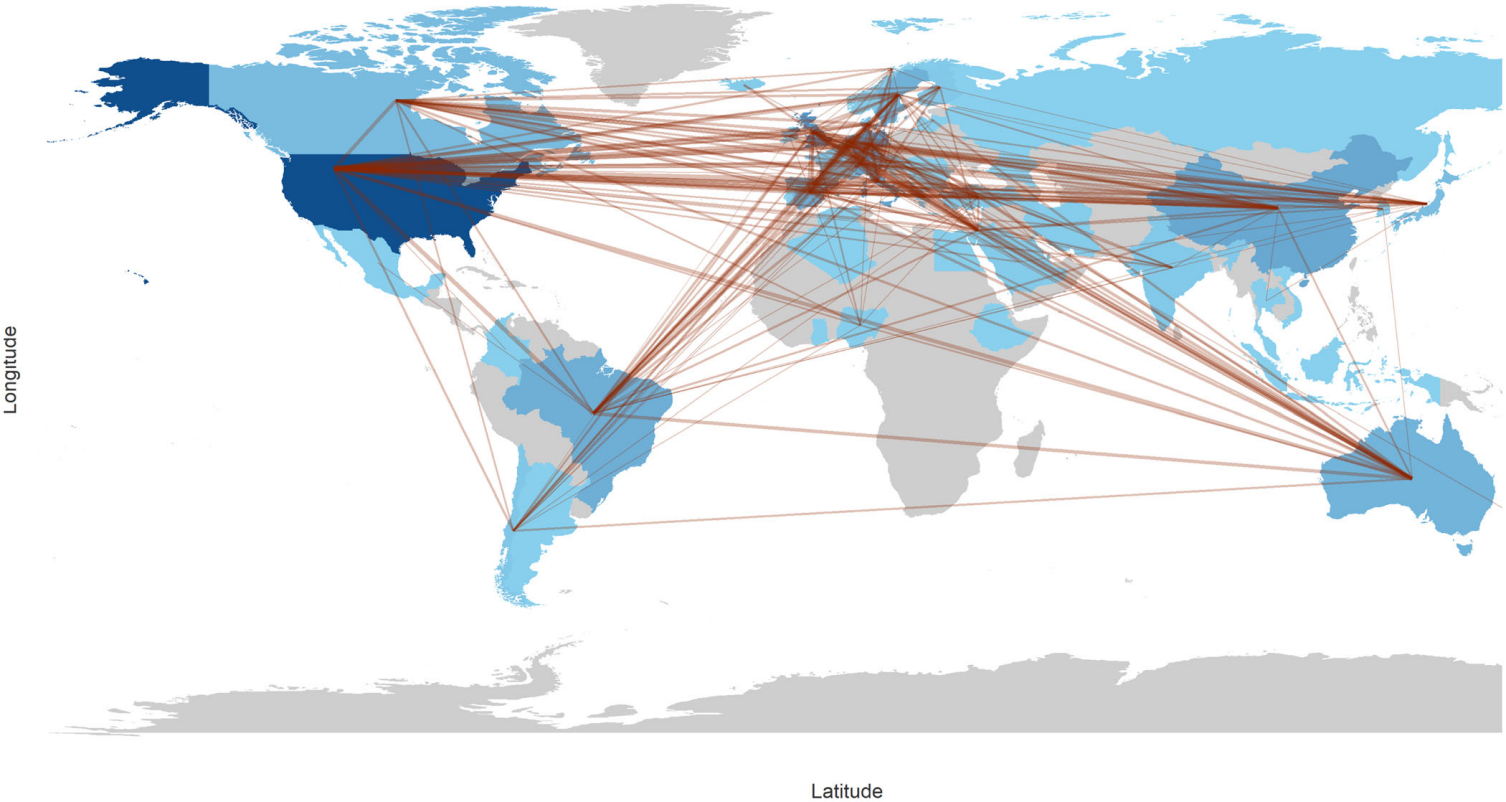
Scientific collaboration between countries.

The study found that authors from 61 countries contributed to the articles, and all of these countries had engaged in scientific collaboration with other countries. Figure 4 illustrates the scientific production of each country, represented by varying shades of blue, as well as the level of scientific collaboration between countries, indicated by the connecting lines.

Based on Figure 4, the intensity of the blue color corresponds to the number of articles, while the lines connecting countries represent scientific collaboration. The countries with the highest level of scientific cooperation are the *United States* and the *United Kingdom* (with 35 articles), the *United States* and *Germany* (with 30 articles), and the *United States* and *Italy* (with 26 articles). Table 2 provides a summary of the countries with the highest number of articles, the greatest level of scientific cooperation, and the most citations.

**Table 2:** Countries with the most articles, scientific cooperation, and citations.

Country	Total link strength	Article	Citations	Norm. citations
USA	374	132	38,620	143.9738
Germany	276	54	15,585	55.7019
England	269	57	19,396	66.6756
Italy	202	44	11,465	48.5813
Switzerland	195	48	14,108	49.1559
Spain	182	34	8745	38.8738
Australia	172	28	8495	32.3216
Brazil	153	33	7744	35.6231
Netherlands	152	28	10,156	32.0073
China	122	33	12,271	47.2624
Sweden	138	30	9775	32.4798

The analysis revealed that the *United States* produced the highest percentage of articles at 21%, followed by *England* and *Germany*, both at 9% each. In terms of citations, the *United States*, *England*, and *Germany* were ranked first, second, and third, respectively.

#### 3.2.3 Institution/University

These articles were produced by authors from 693 universities/institutes. The *University of London* and the *University of Bern* were ranked first and second, respectively, with 30 and 27 articles. The *Complutense University of Madrid* ranked third with 22 articles.

#### 3.2.4 Journal

The findings also showed that 292 articles were published in 55 different journal titles. More than 20% of the articles were published in two journal titles: the *Journal of Dental Research* and *Periodontology 2000*.

#### 3.2.5 Article type and subject category

Out of 292 articles, 269 were published in journals, and 23 were presented at conferences. The findings indicate that 148 articles (50.685%) are original, and 144 (49.315%) are review articles. These articles belong to 10 subject categories, all of which are related to the “Dentistry, Oral Surgery, Medicine” category. It should be noted that some articles are related to two or more subject areas. Thirty articles are related to “Materials Science: Biomaterials” (10.2%).

#### 3.2.6 Web of Science indicators (citation, usage count: last 180 days, and since 2013)

Citation, Usage Count Last 180 Days, and Usage Count Since 2013 are the three indices used to evaluate articles in WoS (Table 3).

**Table 3:** Web of science indicators.

Indicators		Total occurrences	Number of nonzero occurrences	Average number of nonzero occurrences	Min	Max
Citation		77,938	292 (100%)	266.9	10	1848
Usage Count	Last 180 Days	3895	286 (97.9%)	13.6	0	71
	Since 2013	27,851	290 (99.3%)	96.03	0	984

Table 3 shows that all reviewed articles have been cited, and 97.9% of them have been accessed or saved at least once in the last 180 days. The average citation count for the reviewed articles is 266.9. Table 4 displays the articles with the highest citation count.

**Table 4:** Top articles by citation count.

Authors	Document type	Citation	Citation density	180‐day usage count	Since 2013 usage count
Schiffman et al. (2014)	Article	1848	205.3	19	337
Xu et al. (2020)	Article	1563	521	31	174
Ruggiero et al. (2014)	Article	1448	160.9	10	142
Peng et al. (2020)	Review	1102	367.3	25	141
Eke et al. (2012)	Article	1098	99.81	5	160
Kassebaum et al. (2014)	Review	1088	138.2	12	124
Tonetti et al. (2018)	Article	977	195.4	41	153
Marcenes et al. (2013)	Article	970	97	4	93
Meng et al. (2020)	Article	912	304	20	164
Stansbury and Idacavage (2016)	Article	899	128.4	43	984

The result shows that Schiffman et al. (2014) have the highest citation count with 1848 citations. Among the top 10 articles, two are reviews, and eight are original articles. Four out of these 10 articles have been published in the *Journal of Dental Research* (IF = 8.924, CiteScore = 12.10). This journal is ranked in the top 5% of dentistry journals based on SNIP and is included in the first quartile (Q1) in both WoS and Scopus.

Zhu et al. (2020) have the highest usage count in the last 180 days, with 71 occurrences. Among the top 10 articles with the highest usage count, eight are reviews, and two are original articles. Three out of these 10 articles have been published in the International Journal of Oral Science (IF = 24.897, CiteScore  = 24.40). This journal is ranked in the top 1% of dentistry journals based on SNIP and is included in the Q1 category in both WoS and Scopus.

Stansbury and Idacavage (2016) have been cited 984 times since 2013. Among the top 10 articles with the highest usage count, six are reviews and four are original articles. Five out of these 10 articles have been published in Dental Materials (IF = 5.687, CiteScore = 9.2). This journal is ranked in the top 5% of dentistry journals based on SNIP and is included in the Q1 in both WoS and Scopus.

Xu et al. (2020) have the highest citation density with 521. Among the top 10 articles with the highest citation density, one is a review, and nine are original articles. Four of these 10 articles have been published in the *Journal of Clinical Periodontology* (IF = 7.478, CiteScore = 13.5).

#### 3.2.7 Citation links and normalized number of citations

**Figure 5:**
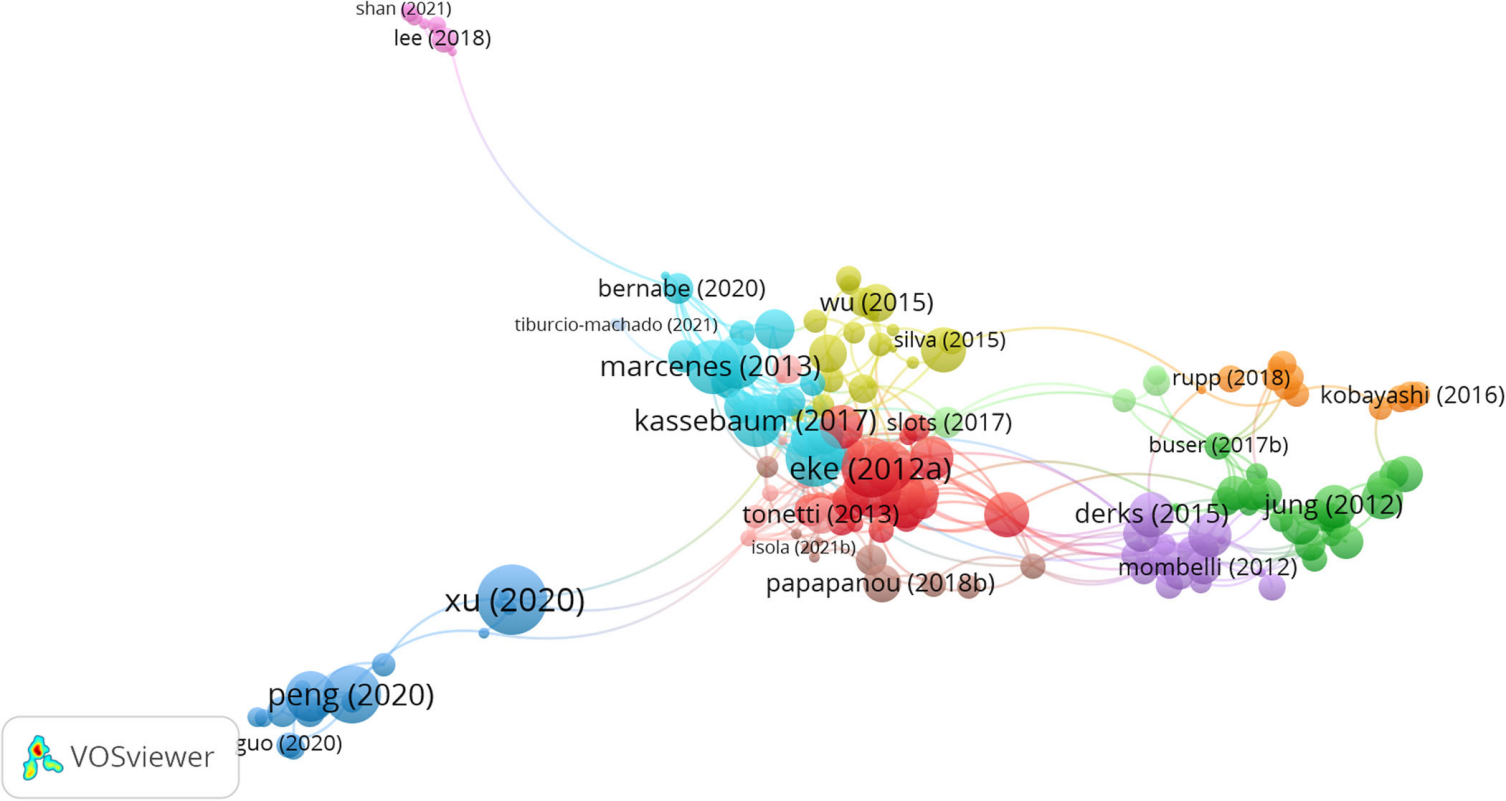
Citation links.

Figure 5 displays the citation links between papers, and Table 3 shows the articles with the highest number of citation links.

According to Figure 5, Kassebaum et al. (2014) have the most citation links with 23 links between articles. This means that this article has a citation link with 23 other articles that are present in the network (cited, citing).

Tonetti et al. (2018) have the highest normalized number of citations with a norm. citations = 3.3433 and 14 citation links. The calculation of the normalized number of citations for a document involves dividing the number of citations received by the document by the average number of citations received by all documents published in the same year and included in the data set used by VOSviewer. This normalization process addresses the difference in citation counts between older and more recent documents, as older documents have had more time to gather citations.

As can be seen in Table 5, articles with the highest number of citation links have been published in the *Journal of Clinical Periodontology* and the *Journal of Dental Research*.

**Table 5:** Top articles in terms of citation links.

Article	Links	Norm. citations
Kassebaum et al. (2014)	23	2.3351
Marcenes et al. (2013)	16	2.5826
Tonetti et al. (2017)	15	1.5871
Marouf et al. (2021)	15	1.7566
Tonetti et al. (2018)	14	3.3433
Caton et al. (2018)	14	2.2746
Schwarz et al. (2018)	14	1.5082
Sanz et al. (2020)	14	1.1484
Sanz et al. (2018)	12	0.8789

#### 3.2.8 Keywords co‐occurrence map and abstract word cloud

**Figure 6:**
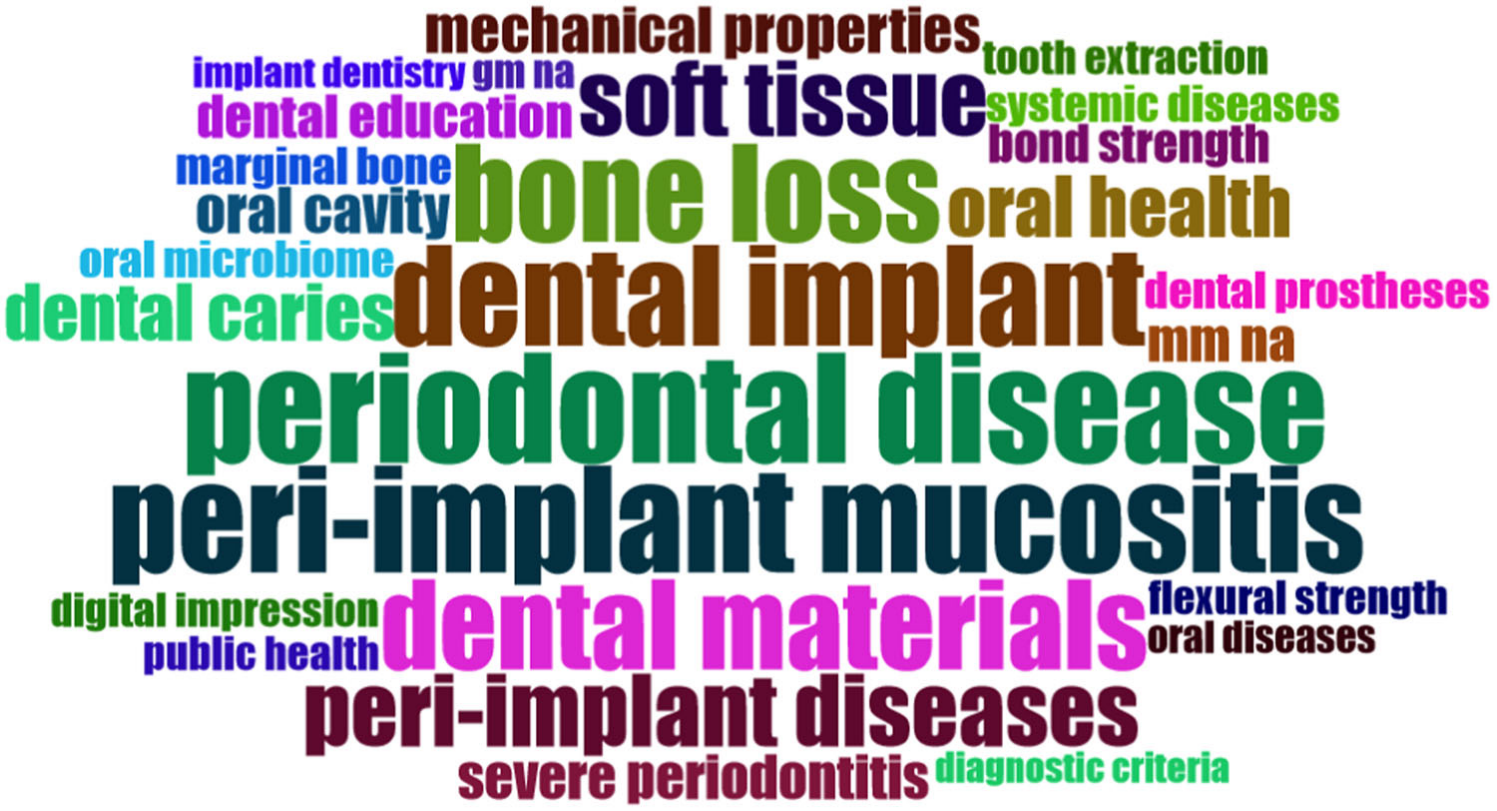
Abstract word cloud.

A word cloud is a visualization of word frequency in a text, where the font size of each word is determined by its prevalence in the text (Figure 6).

**Figure 7:**
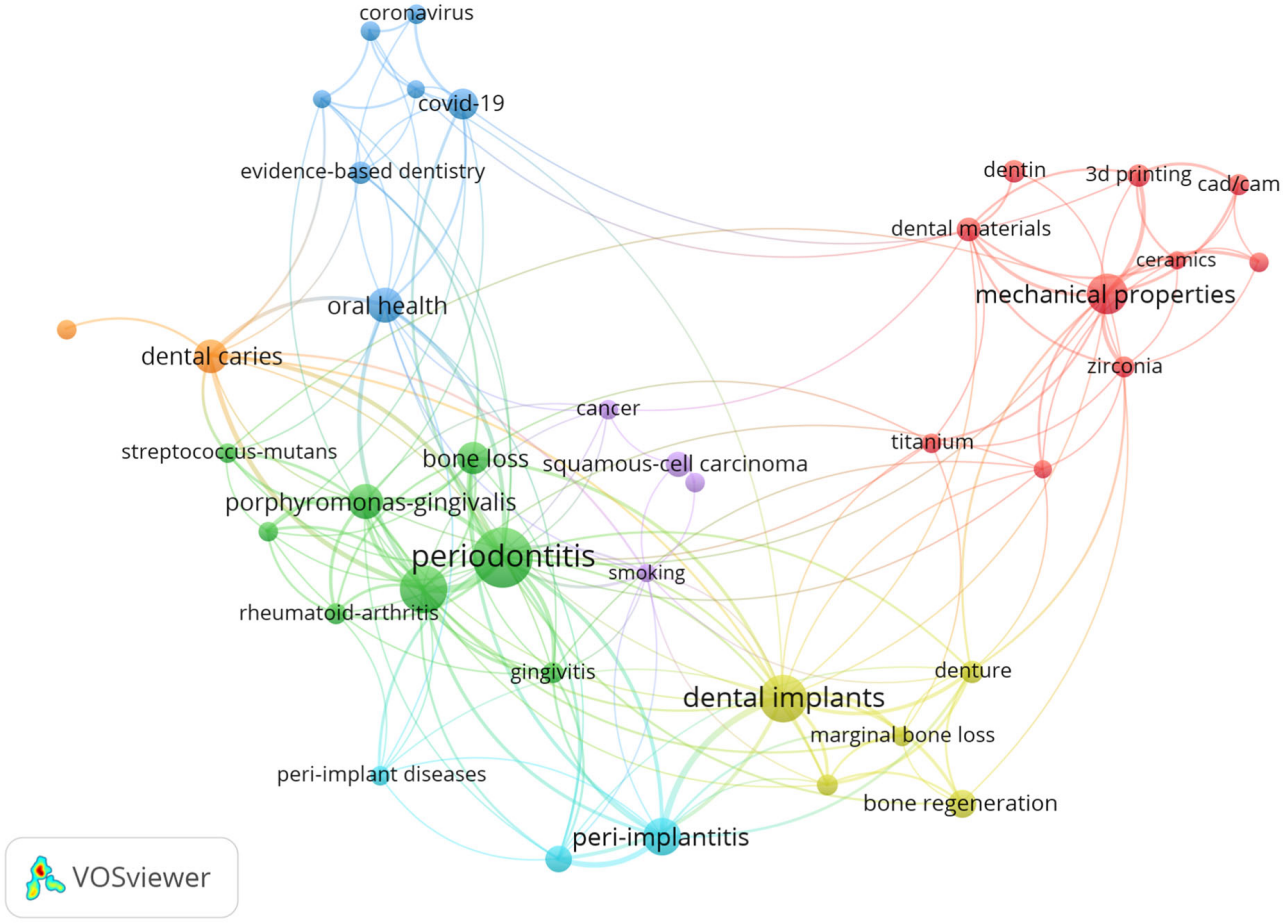
Keyword co‐occurrence map.

In the keywords co‐occurrence map, the size of the circles indicates the frequency of occurrence and the level of existing knowledge for each concept. The nodes represent concepts, and the lines depict their relationships (Figure 7).

According to the keywords co‐occurrence map and abstract word cloud, the most frequent keywords are “periodontitis,” “dental implants,” “periodontal disease,” “mechanical properties,” “peri‐implantitis,” “oral health,” “dental caries,” “dental materials,” and “3D printing.” Other commonly used keywords include “Porphyromonas gingivalis,” “bone loss,” “COVID‐19,” “peri‐implant mucositis,” “evidence‐based dentistry,” “dental education,” and “cancer.”

## 4 DISCUSSION AND CONCLUSION

The findings showed that 495,648 articles in dentistry were indexed in WoS. The *University of London* had the highest number of articles, followed by the *Universidade de Sao Paulo* and the *University of California System*. The *United States*, *England*, and *Brazil* produced the highest number of articles, which were published in 692 journals. The *Journal of Dental Research* had the highest number of publications. Out of 495,648 articles, 292 top papers were identified, with 1341 authors participating. *Mariano Belén Sanz* and *Maurizio S. Tonetti* were the top two authors. The *University of London* and the *University of Bern* had the highest number of publications. The *United States* produced the highest number of articles and had the most scientific collaborations with other countries. The top 292 articles were published in 55 journals, with over 20% of them appearing in the *Journal of Dental Research* and *Periodontology 2000*. Wiley had the highest percentage of articles at 42.4%, while *Elsevier* came in second place with 29.1%. The 292 reviewed articles were cited 77,938 times, with an average of 266.9 citations per article.

According to the keyword co‐occurrence analysis, the most frequent keywords in the field of dentistry include “periodontitis,” “dental implants,” “periodontal disease,” “mechanical properties,” “peri‐implantitis,” “oral health,” “dental caries,” “dental materials,” “3D printing,” “Porphyromonas gingivalis,” “bone loss,” “COVID‐19,” “peri‐implant mucositis,” “evidence‐based dentistry,” “dental education,” and “cancer.” Some bibliometric research has been conducted on various aspects of these topics. One study evaluated 117 publications from 2003 to 2022 and identified a global research trend on “photodynamic therapy in periodontitis.” The results showed that Brazil was the leading country in this research area, while all the significant contributors were organizations from the United States. The *Journal of Periodontology* published the highest number of papers, which received a high number of citations (Al‐Khalifa et al., 2023). Another study found that between 1980 and 2010, 1794 documents were published on “regenerative periodontal surgery,” with the Journal of Periodontology and the Journal of Clinical Periodontology being the primary journals in this field (Gutiérrez‐Vela et al., 2012).

Geminiani et al. (2014) demonstrated that a total of 2076 documents were published on periodontal disease in five peer‐reviewed journals between 1995 and 2010. Research on periodontal disease has undergone significant changes over the past 15 years. The studies were categorized into periodontal disease, mucogingival surgery, dental implants, and other topics. Research articles that examined the anatomical aspects of the periodontium, classification systems, epidemiological patterns, causes, diagnostic methods, treatment approaches, maintenance strategies, or long‐term outcomes of periodontal disease were classified under the category of “periodontal disease.” Dental implant‐related topics are becoming increasingly prominent in periodontal research. Shaikh et al. (2019) demonstrated that the most frequently cited articles in the field of periodontal regeneration concentrate on topics such as “guided tissue regeneration,” “intrabony periodontal defects,” “enamel matrix derivatives,” and “platelet‐rich plasma.”

The top 100 publications on “periodontal disease” primarily focus on the correlation between periodontal disease and cardiovascular disease and diabetes mellitus. Subsequently, an investigation was conducted into the systemic complications associated with periodontitis, including its effects on pregnancy, rheumatic, pulmonary, and cerebrovascular diseases, as well as cancer. The main keywords employed in this study were “periodontal disease,” “periodontitis,” “inflammation,” “diabetes mellitus,” and “atherosclerosis” (Ahmad et al., 2020).

A study was conducted to assess 476 articles on the impact of diabetes on oral implant treatments using bibliometric analysis. The findings revealed that Clinical Oral Implants Research had the highest number of articles, while the Journal of Clinical Periodontology had the highest number of citations (Lorusso et al., 2020). From 2008 to 2013, a study found 6088 articles on implantology. The most frequently used keywords were “implant,” “bone,” and “prosthesis.” “Implant failure” was also frequently mentioned. The study focused on survival and complication rates, which are crucial for improving treatment outcomes, reducing costs, and enhancing patient satisfaction (Tarazona et al., 2017).

Tarazona‐Álvarez et al. (2021) analyzed 2547 scientific publications on peri‐implant diseases. The most productive institutions in this field were the *University of Bern*, *Gothenburg University*, and the *University of Michigan*. The most frequently used keywords were “periimplantitis,” “dental implant,” and “peri‐implant mucositis.” The most frequently used keywords in publications on treatment included “treatment,” “antimicrobial,” “implantoplasty,” “surgical therapy,” “air abrasion,” “maintenance,” “guided bone regeneration,” “decontamination,” “laser,” “bone regeneration,” and “chlorhexidine.”

Another study aimed to utilize bibliometric indicators to identify trends in digital implant dentistry. A total of 3680 publications were analyzed. The *University of Bern* emerged as the most productive institution, followed by the University of Sao Paulo and the University of Michigan. Research on digital workflow, digital impressions, and 3D printing is gaining popularity. The most commonly discussed topics in these articles are treatment outcomes, accuracy of digital technology, planning and placement of implants, radiography and anatomy, and implant stability and biomechanics (Chen et al., 2020). A study conducted to identify classic articles in implantology found that research on complications, peri‐implant infection, pathology, and therapy had increased in production over time (Chiang et al., 2018). Other studies have evaluated topics such as prosthetic complications associated with dental implants and have identified the trends of the top 100 cited articles on peri‐implantitis research (Khan et al., 2023).

Dental caries is another important topic in the field of dentistry that has been the subject of bibliometric analysis. The analysis of author keywords revealed that the terms “dental caries,” “caries detection,” “caries,” and “diagnosis and radiography” were the most commonly co‐occurring terms (Ganesh et al., 2023). Analysis of bibliometrics on studies related to root caries reveals that the United States has been the leading country in producing research publications in this field from 1992 to 2021. The Journal of Dental Research and Caries Research are the main journals in this field. The University of London has had the highest number of publications in the last 30 years. The commonly used keywords in the past three decades include “demineralization,” “remineralization,” “aged,” “dentin,” and “fluoride” (Ji et al., 2023).

A comprehensive analysis of 934 research studies on the utilization of three‐dimensional (3D) printing in the field of dentistry revealed that the primary purpose of 3D printing technologies is to assist traditional dental procedures. The increasing prevalence of 3D printing technologies within the dental profession can be attributed to the escalating refinement of their applications (Balhaddad et al., 2023). A bibliometric analysis was conducted on a data set of 3324 papers about oral health‐related quality of life. Through the process of analyzing keywords, several significant topics have surfaced. A bibliometric analysis was conducted on a data set of 3324 papers related to oral health and quality of life. Through the process of analyzing keywords, several significant topics have emerged. These include the development and validation of measurement instruments, research specifically targeting the younger population, as well as clinical investigations on operative dentistry, implantology, orthodontics, and community dentistry (Yu et al., 2023).

Additionally, the number of citations a research paper receives can be influenced by various factors, such as the author's reputation, the journal of publication, and the country of origin. Although citation count is used to assess the impact of an article, it does not necessarily correlate with its quality. Nonetheless, it can be considered as a measure of academic impact (Nieminen et al., 2006). The highly cited topics in dentistry include “periodontitis,” “dental implants,” “periodontal disease,” “mechanical properties,” “peri‐implantitis,” “oral health,” “dental caries,” “dental materials,” “3D printing,” “Porphyromonas gingivalis,” “bone loss,” “COVID‐19,” “peri‐implant mucositis,” “evidence‐based dentistry,” “dental education,” and “cancer.” Therefore, reviewing the highly cited articles on dentistry can provide insight into the topics that have captured the interest and attention of researchers in the field of dentistry. The interest and attention of researchers also originate from the needs of society and patients. The findings of this study have significant utility for researchers in the field of dentistry, serving as a valuable resource for self‐evaluation and the development of future research strategies.

Assessment of researchers' work is now more dependent on publication output and citation impact metrics than on peer review (Cameron, 2005; Haustein & Larivière, 2014). The use of bibliometric indicators is increasingly common among governments and funding organizations due to their scalability, lower costs, and perceived objectivity. The aim is to optimize research allocations and make funding more efficient (Haustein & Larivière, 2014; Weingart, 2005). The bibliometric analysis provides an overview of the literature in a specific field and is frequently used as the basis for making research funding decisions (Ellegaard, 2018).

The results of this study enable readers to identify the authors, institutions, countries, and journals that make the greatest contributions to the compilation of highly cited dental articles. The results of this study can also provide valuable information on highly cited dental articles, including their usage and citation density indicators. In addition, the study presents the level of scientific collaboration among authors and countries in compiling highly cited dental articles. Moreover, bibliometric citation analysis has been extensively employed in the field of medicine to assist researchers in gaining a comprehensive understanding of the subject and identifying specific areas of popular interest for further investigations.

This study has some limitations. In this study, we reviewed highly cited dental papers. Highly Cited Papers are defined as those that rank in the top 1% by citations for the specific field and publication year in the WoS. These data are derived from ESIs. It is suggested to evaluate highly cited dental articles indexed in Scopus for future studies. Additionally, this article has addressed the scientific impact of dental articles, or, in other words, the number of article citations. It is suggested to investigate the social impact (views, downloads, shares, tweets, likes, and dislikes) of these articles on social networks like Twitter, Facebook, and YouTube.

## AUTHOR CONTRIBUTIONS

**Amene Taghdisi Kashani**: Conceptualization; data curation; supervision; validation; visualization; roles/writing—original draft; writing—review and editing. **Zahra Batooli**: Conceptualization; data curation; formal analysis; funding acquisition; investigation; methodology; project administration; software; supervision; validation; visualization; roles/writing—original draft, writing—review and editing. **Mostafa Mozafari**: Data curation; formal analysis; investigation; roles/writing—original draft; writing—review and editing.

## CONFLICT OF INTEREST STATEMENT

The authors declare no conflict of interest.

## ETHICS STATEMENT

All procedures performed in studies were in accordance with the ethical standards of the Declaration of Helsinki. The study has been approved by Kashan University of Medical Sciences Ethical Committee (IR.KAUMS.MEDNT.REC.1402.037).

## Data Availability

Data will be made available on request.
